# The Hospital World

**Published:** 1911-06-10

**Authors:** 


### The Hospital World.

### A Governor's Bequest to Pendlebury.

The late Vernon K. Armitage, Esq., M.A., J.P., who had been a member of the Board of Governors of the Manchester Children's Hospital, Pendlebury, for twenty-six years, has bequeathed a legacy of £1,000 for the general purposes of the hospital.

### Bequests to some Gloucester Hospitals.

The will of the late Right Hon. Sir John Edward Dorington, Bart., of Lypiatt Park, Stroud, and Queen Anne's Gate, is now known. He left £278,989 gross, and has bequeathed £500 to the Stroud Hospital and £200 to the Gloucester Infirmary. The Royal West of England Sanatorium at Weston-super-Mare receives £50, and the Westminster Hospital £100. Sir John was formerly M.P. for Stroud and North Gloucestershire, and was in his seventy-ninth year.

### Sequel to the Savoy Ball.

The recent gathering at the Savoy in fancy dress in aid of the Prince Francis of Teck Memorial Fund of the Middlesex Hospital has had a graceful sequel. On the next Friday Prince and Princess Alexander gave a reception in the same ballroom to those who had worked so hard to make the ball itself a success. Prince Alexander specially drew attention to the services of Lady Gerard and Mrs. Hwfa Williams, both of whom were among those who sold tickets and took parties. The directors of the hotel and the firms who contributed gifts or prizes were thanked also by the Prince, who must have been greatly encouraged by the total contribution of £2,000, which the ball has been the means of raising for the funds of the Middlesex Hospital.

### The New Secretary of St. Bartholomew's Hospital, Rochester.

The Secretaryship of the ancient foundation of St. Bartholomew's Hospital, Rochester, has fallen to Mr. Chas. Speyer, who has seen eleven years' service in St. Mary's Hospital. Mr. Speyer entered as a clerk, and has been promoted to the office of steward; from this he takes up his duties in the Cathedral City. He is 31 years of age, and we wish him success on his promotion. It is worthy of note that the provincial hospitals are following the lead of those in London and requiring that the affairs of their institutions shall be placed in the hands of trained men. There have been too many failures through hospitals being directed by men who knew little of the intricacies of a hospital career. That has been the case in the past, but it is more than ever necessary now that men of experience be appointed to meet the exacting demands of the hospitals of to-day.

